# Mr. Lipscomb, on Dyspnea

**Published:** 1800-06

**Authors:** G. Lipscomb

**Affiliations:** Surgeon, at Birmingham


### To the Editors of the Medical and Physical Journal.

GENTLEMEN,

THE very laudable design of your excellent Publication being to rescue the practice of medicine from the hands of empirics, by encouraging a recital of such cases as may be conducive to the improvement of science, either by tending to establish just theory, or to refute vague hypothesis, I do myself the honour of transmitting the following account of a patient who was placed under my care in the year 1798; and, if it shall be thought of sufficient importance to merit a place in your Journal, the insertion of it will oblige,

GENTLEMEN,

Your most humble servant,

G. LIPSCOMB,

*March* 6, 1800. Surgeon, at Birmingham.

---

### CASE.

William Hunt, of St. Nicholas' Parish, Warwick, aged thirty years, had, during almost two years, been afflicted at irregular intervals with a difficulty of breathing, which came on more frequently in the evening when he lay down in bed; was succeeded by excruciating pain about the heart; and when the pain went off, great faintness and weakness followed. His appearance was emaciated, his skin tinged with a yellowish cast, and the smallest exertion brought on the difficulty of respiration. He slept very unquietly, and the pain, which commenced at the scrobiculus cordis, extended up towards his shoulders, particularly on the left side. His pulse was hard and oppressed, varying considerably when the breathing became affected; it then usually beat with amazing celerity, and was greatly laborious. As soon as the difficulty of respiration ceased, the pulse sunk rapidly, till it was scarcely perceptible. It has often decreased in frequency, while I kept my fingers on the radial artery, from 160 beats in a minute to 40. It continued very slow while the pain lasted, and gradually acquired its accustomed degree of celerity when that symptom ceased. The pain and oppressed breathing *seldom* took place at the same time; the former *almost always* succeeded the attack of the latter; and the duration of the paroxysm varied from a few minutes to three hours or longer. The urine was high coloured, and deposited a reddish flocculent sediment: it was less turbid when evacuated, either in the paroxysm or soon afterwards; wards; and its quantity was confiderable. The ftate of the bowels was regular, but the pain returned more frequently when a diarrhœa had fupervened. The fkin was dry, but eafily relaxed; and the pain was mitigated when a copious perfpiration took place; the extremities were *very* cold during the paroxyfms.

The unfortunate fubject of thefe diftreffing complaints, previous to his being placed under my care, had confulted *feveral* practitioners, and taken a great variety of medicines without any confiderable benefit. The wheezing and oppreffion of the cheft, when he lay down, induced me to fufpect, that the functions of the lungs were interrupted by the prefence of a fluid in the cavity of the thorax; but the ftricteft examination did not afford the fmalleft proof in fupport of that conjecture, for the thorax was not at all enlarged; and when he affumed a vertical pofition, he could walk about brifkly without *any* inconvenience. The ftate of the pulfe induced me to bleed him; twelve ounces of blood were taken away; and the *three* enfuing paroxyfms were fhorter and lefs painful than ufual. His pulfe became fofter and lefs obftructed, and in the paroxyfm did not increafe to more than 110. The operation was repeated in a few days, but without any apparent advantage. The blood coagulated fpeedily; the proportion of red particles was but fmall, and the ferum was turbid, refembling pus diluted with water: the violence of the pain and the difficulty of breathing again increafed. The fecretion of urine was promoted by diuretics, without much advantage; powder of digitalis was continued for a fortnight without any benefit. He complained of great pain in the kidneys, and evacuated fome gravel: the pain continued, and the diuretics were laid afide. A fort of afthmatic paroxyfm took place, which was relieved by ammoniacum fquills and other expectorants, a very thick and vifcid phlegm being thrown up. His appetite was much impaired, and he flept little. Naufeating dofes of ipecacuanha joined with calomel were given, but no beneficial alteration followed the ufe of any medicine whatever. He was feen by different practitioners with whom I happened to be acquainted; and in April, (about two months after he had been placed under my care) one of them ftrongly recommended the ufe of guaiacum, which was accordingly exhibited in large dofes, but with no advantage. The pain in the paroxyfm became intolerable, and opium was reforted to from neceffity; for, as I had long thought there was a confiderable derangement of the heart or the great veffels, it did not appear to me at all probable, that antifpafmodics would produce benefit. Opium was given in dofes of 2, 3, 4, and 5 grains, at the commencement mencement of the paroxyfm, without any diminution of the violence of the pain or its continuance. Blifters were applied to the fcrobiculus cordis, which difcharged plentifully, but the pain continued to recur at fhort intervals. No particular alteration took place, excepting that the patient's appetite was a little increafed by the ufe of infufion of quaffia, until the 9th of May, when he complained of a total fuppreffion of urine; and the next morning, as he was walking acrofs his room, he fell down fuddenly and expired.

The relations of the deceafed very readily fubmitted to my requeft to be permitted to examine the ftate of the vifcera; and the appearances, on diffection, were as follow:

The lungs were found diminifhed to lefs than half of their natural fize, pale, flaccid, the inferior edges of both lobes difcoloured and apparently impervious. No tubercles, nor matter in the bronchia; more than five pints of a pale watery fluid in the cavity of the thorax. The pericardium, greatly diftended, contained one pint (exactly) of the fame fluid. The heart confiderably enlarged. The left ventricle full of coagulum. The right contained a large polypus, part of which paffed into the auricle, and prevented the valves being applied clofe. The end, which terminated in three points, reached about three inches into the vena cava. The pulmonary artery was in a natural ftate.

The liver was difcoloured, but firm and free from tubercles. The gall bladder of a large fize, and full of bile. The duodenum in a natural ftate. The jejunum, ileum, and colon of a darker colour than ufual. The bladder empty, though no urine had been difcharged for more than twenty-four hours. The appendic. verm. was not quite half an inch long. The omentum was alfo remarkably fmall. The fpleen and kidneys afforded no uncommon appearance.

The diminution of the fize of the lungs feemed to have been as gradual as the accumulation of the water; and this accounts for no fluctuation being perceptible, nor any degree of tumefcence obfervable, during the progrefs of the difeafe.

After the death of the patient (but not before) his relations recollected that he had fuftained an attack of peripneumony about the time when the complaint of difficulty of breathing firft took place, in confequence of immerfion into the river Avon on a very hot day.

In the relation of fuch cafes as the prefent, humanity cannot but fhudder at the deficiency of the medical art. We fee difeafe baffle the moft induftrious exertions, and we feel the infufficiency of human acquirement. For if the underftanding can develope the *caufe*, the hope of relief is but farther removed from us, and we *know* only to *lament!*

